# Nitrogen Removal from Domestic Wastewater and the Development of Tropical Ornamental Plants in Partially Saturated Mesocosm-Scale Constructed Wetlands

**DOI:** 10.3390/ijerph16234800

**Published:** 2019-11-29

**Authors:** Carlos Nakase, Florentina Zurita, Graciela Nani, Guillermo Reyes, Gregorio Fernández-Lambert, Arturo Cabrera-Hernández, Luis Sandoval

**Affiliations:** 1 Division of Research, Postgraduate Studies and Innovation, Tecnológico Nacional de México/Instituto Tecnológico Superior de Misantla, Misantla, Veracruz C.P. 93821, Mexico; 2 Quality Environmental Laboratory, Centro Universitario de la Ciénega, University of Guadalajara, Ocotlán, Jalisco C.P. 47820, Mexico; 3 Department of Engineering in Business Management, Tecnológico Nacional de México/Instituto Tecnológico Superior de Misantla, Misantla, Veracruz C.P. 93821, Mexico; 4 Master of Engineering in Tecnológico Nacional de México/Instituto Tecnológico Superior de San Andrés Tuxtla, San Andrés Tuxtla, Veracruz C.P. 95804 Mexico

**Keywords:** constructed wetlands, nitrogen, ornamental plants, biomass, wastewater

## Abstract

Vertical partially saturated (VPS) constructed wetlands (CWs) are a novel wastewater treatment system for which little information is known about its design parameters and performance under tropical climates. The objective of this study is to evaluate the nitrogen removal process from domestic wastewater and the production of tropical ornamental plants (*Canna hybrids* and *Zantedeschia aethiopica*) in VPS CWs at a mesocosms scale. Nine VPS CWs, with a free-flow zone of 16 cm and a saturated zone of 16 cm, were used as experimental units. Three units were planted with *Canna hybrids.,* and three, with *Zantedeschia aethiopica* (one plant per unit); the remaining three units were established as controls without vegetation. They were fed with domestic wastewater intermittently and evaluated for the elimination of COD, N-NH_4_, N-NO_3_, Norg, NT, and PT. The results showed an increase in the removal for some pollutants in the vegetated systems, i.e., N-NH_4_ (35%), Norg (16%), TN (25%), and TP (47%) in comparison to the unvegetated systems. While N-NO_3_ removal showed better removal in 10% of the systems without vegetation, no significant differences were found (*p* > 0.05) for COD removal. The aerobic and anaerobic conditions in the VPS CWs favor the elimination of pollutants in the systems, and also the development of the tropical species evaluated in this study; good development was exhibited by a high growth rate and biomass production.

## 1. Introduction

Nitrogen is a common pollutant present in wastewater around the planet [1,2,3]. Its presence in a range of concentrations in wastewater that are discharged without treatment or poorly treated in surface aquatic bodies provokes negative impacts in aquatic ecosystems such as rivers and lakes [4]. Nitrogen is one of the main pollutants responsible for eutrophication of surface waters that in turn reduces dissolved oxygen and endangers aquatic life. Nitrogen concentrations in surface water and groundwater bodies are difficult to regulate and control [5,6]. In wastewater, nitrogen can be present in different forms and can be transformed when wastewater is treated by different mechanisms. In the process of nitrification and denitrification, nitrogen suffers two transformations if it is in the form of ammonium. The first is nitrification, where ammonium is converted to nitrate and develops in the presence of oxygen. However, through nitrification, nitrogen is not removed from the wastewater; it only changes chemically. In the second step, in denitrification that develops in an anoxic/anaerobic environment, nitrate is transformed into a removable gaseous product [7,8]. According to Metcalf and Eddy [9] and Bojorges et al. [10], in domestic wastewater, nitrogen is present as organic nitrogen (Norg), ammonium (N-NH_4_), and nitrate (NO_3_+). A cost-effective natural alternative to conventional wastewater treatment is constructed wetlands (CWs), which are engineered systems that mimic the functions of natural wetlands in terms of their phytoremediation capacity [11,12]. These are composed of substrates, plants, microorganisms, and wastewater and can be designed to remove nitrogen present in wastewaters with favorable results; although their elimination in CWs along with phosphorus is still a challenge [13]. In this sense, new design trends have been developed by combining different types of wetlands in a single system to achieve higher removal efficiencies of these pollutants [14,15], taking into account the transformation mechanisms in wetlands. For the elimination of total nitrogen, Norg must be transformed to N-NH_4_+, increasing the concentration of that already present in the wastewater. N-NH_4_ can be assimilated by plants through their roots or by aerobic and anaerobic microorganisms that are present in the systems transforming it back into Norg [16]. In addition, at pH > 8, ammonium is transformed into ammonia and might be volatilized or sequestered on the substrates by ion exchange [17,18]. However, the main mechanism of elimination of N-NH_4_ in CWs is the conversion to NO_3_- in aerobic conditions by *Nitrosomonas* and *Nitrobacter* [16,19]. Finally, the elimination NO_3_- in CWs takes place by the assimilation of plants in their tissues by absorption through the radical zone, but mainly through denitrification that is possible in anoxic/anaerobic conditions, where the nitric oxide (NO) is transformed to nitric oxide (N_2_O) and finally to nitrogen gas (N_2_) [20,21,22]. The different mechanisms for transforming nitrogen in wetlands depend on the type of wetland. Those with a horizontal subsurface flow are constantly saturated, prevailing anoxic/anaerobic conditions that favor denitrification (if organic matter is present), while in those with vertical flow, the presence of oxygen is greater, given their intermittent feeding, so nitrification is favored [10,20]. Although combined systems (vertical and horizontal flow wetlands) have been used to solve this problem, they increase the construction and implementation costs, as well as the space required for their operation [23,24]. An alternative to properly remove nitrogen is to include saturated and unsaturated conditions in a system in the same cycle, such as partially saturated vertical wetlands [13,25,26,27]; nitrification in these systems is possible in the free-flow zone and denitrification in the saturated zone of the system. Unfortunately, information on the operating mechanisms and the installation of these systems is little and are not clearly understood. In order to provide information on the design parameters and performance of these systems in tropical climate conditions, this study aims to evaluate the process of nitrogen removal present in domestic wastewater and the production of tropical ornamental plants (*Canna hybrids* and *Zantedeschia aethiopica*) in vertical partially saturated (VPS) constructed wetlands (CWs) at a mesocosm scale.

## 2. Materials and Methods

### 2.1. Description of the Study Area

This study was conducted at the facilities of Instituto Tecnológico Superior de Misantla (ITSM), Misantla, Veracruz Mexico, from 15 January 2019 to 15 July 2019. Misantla is a municipality that has a tropical climate, with an average annual temperature of 23.3 °C; the coldest month of the year is December, and the hottest is June with temperatures of 19.9 °C and 32 °C, respectively; the average annual rainfall is 1862 mm [28]. The systems were protected by a shadow mesh (50% shade) at the height of 3.5 m from the ground. The wastewater used for the performance of this study was taken from a municipal sewer line which passes through the ITSM facilities. The wastewater was collected with a pump (Truper 1-HP-Model: BOAP-A) and was stored in a tank of 1500 L, which worked not only for storage but also to provide sedimentation to the municipal wastewater whose physico-chemical composition is shown in Table 1.

### 2.2. Description of the System

**Figure 1:**
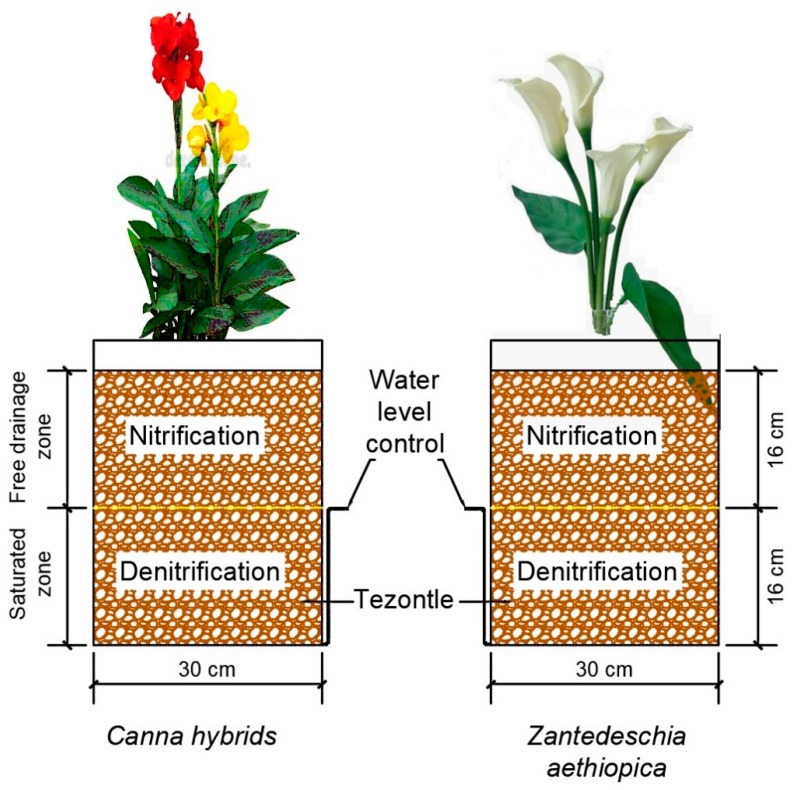
Configuration of the Vertical partially saturated (VPS) constructed wetlands (CWs) at a mesocosms scale.

Nine experimental units at mesocosm level were constructed in cylindrical units of high-density polyethylene and high molecular weight (recycled from paint containers) with a volume of 19 L. The experimental units imitated the operation of VPS CWs and were exposed to the environmental conditions but protected from direct sunlight by a 50% shade mesh. The wastewater level in all systems remained 16 cm below the surface of the substrate, generating a zone of constant saturation (anoxic/anaerobic), and the remaining 16 cm were of a drainage-free zone (Figure 1).

Tezontle was used as a substrate, with a diameter of 1 to 3.5 mm and a measured porosity of 0.53. This material has a larger contact surface than basaltic rocks, although it has the same chemical composition [29]. It is an inert material that has no toxic substances and is physically stable; its pH is close to neutral [30] and it is easily found in the study area.

The mesocosms were fed intermittently with 160 mL every 2 h by automatic 12-W pumps. Ornamental species of tropical climates were used, i.e., *Canna hybrids* and *Zantedeschia aethiopica*, which were collected from their natural habitat close to the area where the experimental units were established. All plants had a height in the range of 15 to 20 cm. Three VPS CWs mesocosms were planted with *Canna hybrids* and three with *Zantedeschia aethiopica* and other three units filled with the same substrate but without vegetation were used as controls. The systems were fed for two weeks with tap water and during the following three weeks, with diluted wastewater until 15 January 2019; thereafter, the systems were fed by wastewater directly.

### 2.3. Plant Development

In order to know the influence of different parameters on plant development, when the plants were well established in each mesocosms, the number of shoots, flower length, leaf length, leaf width, stem thickness, and plant height were measured at 60, 120, and 180 days. The production of flowers was also quantified each month during the study period.

### 2.4. Biomass Production

Once the experiment was finished, the plants (*n* = 6 for *Canna hybrids*, and *n* = 6 for *Zantedeschia aethiopica*) were removed to determine the aerial and underground biomass, for which the plants were separated from its root. Each section of the plants was washed and dried in the open air for 48 h and then placed in an oven at 100 °C for at least 72 h to obtain its constant weight. Finally, to obtain the biomass production, the plants were weighed by a digital analytical balance (Shimadzu AUW-220D) [31].

### 2.5. System Monitoring

Once the VPS CWs mesocosms were stabilized, the wastewater quality parameters were measured at both mesocosm inputs and outputs from 15 January 2019 to 15 July 2019, every 15 days using standard methods for wastewater analysis [32]. These parameters were dissolved oxygen (DO), water temperature, pH, total phosphorus, chemical oxygen demand (COD), ammonium (N-NH_4_), nitrates (N-NO_3_), and total nitrogen Kjeldhal (TNK).

### 2.6. Data Analysis

The results obtained from the removal of contaminants were analyzed with the Dunnett test. The equality of means between control and vegetated systems was established as a null hypothesis, with a 95% confidence interval as a statistical requirement of this test. The data independence test was developed referring to X^2^ of Bartlett variance test [33] in Statistical Software R version 3.6 and RStudio 1.1.4.

## 3. Results

### 3.1. Plant Development

**Figure 2:**
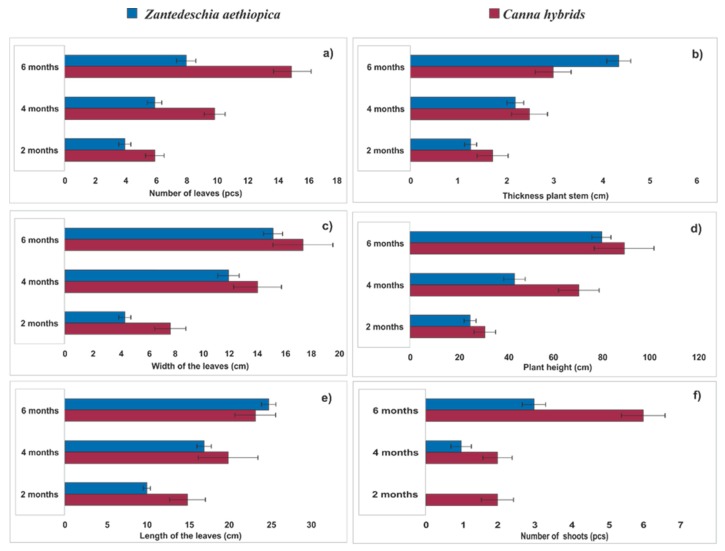
Development of *Canna hybrids* and *Zantedeschia aethiopica*, during the period of study in VPS CW mesocosms. (**a**) Number of leaves, (**b**) Thicness plant stem, (**c**) Width of the leaves, (**d**) Plant height, (**e**) Length of the leaves, (**f**) Number of shoots. Average ± standard deviation.

The growth of *Canna hybrids* and *Zantedeschia aethiopica* was monitored bimonthly for six months based on various vegetative growth parameters including plant height, stem thickness, number of leaves, leaf width and length, as well as the number of shoots. The sustained and vigorous growth of both species was observed according to the different parameters (Figure 2).

The *Canna hybrids* reached a value of 90 ± 10 cm in height at the end of the experiment (Figure 2d), with a stem diameter of 3 cm ± 0.5 cm; these values are similar to those reported for several varieties of *Canna* spp., in experimental agricultural fields under optimal growing conditions. For *Zantedeschia aethiopica*, the average maximum height was 80 cm ± 5 (Figure 2d) with a stem diameter of 4.4 ± 0.5 cm; these values are congruent with those reported by Cruz-Castillo and Torres-Lima [34] in the crops of this species in producing areas within the same state of Veracruz, where this study was carried out.

**Figure 3:**
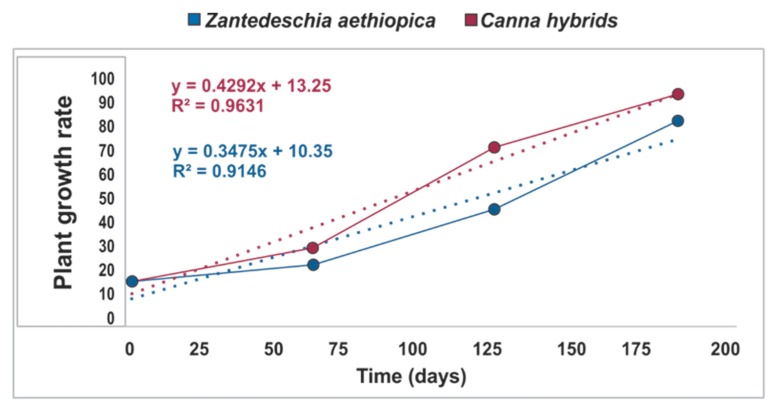
A linear relationship between the height increase and time for the two species.

On the other hand, the increase in the height of the two species showed a linear relationship with respect to time. For *Canna hybrids* and *Zantedeschia aethiopica* (Figure 3) the height increases were 0.4166 cm/day (R^2^ = 0.9631) and 0.388 cm/day (R^2^ = 0.9146), respectively. This information is congruent for the cultivation of the same species in other CW systems reported by other authors [35,36]. Additionally, the value of the leaf area was estimated by applying the equation proposed by Kato et al. [37]: Y = 0.704X; where *Y* is the leaf area and *X* is the product of the length multiplied by leaf width (Figure 2a,c,e). It was found that the leaf areas for *Canna hybrids* were 475, 1971, and 4128 cm^2^ for 2, 4, and 6 months, respectively; while for *Zantedeschia aethiopica*, these were 112, 861, and 2112 cm^2^, respectively. These values are lower than those reported in agricultural crops of the two species [38]. Similar to the increase in height, the increase of the leaf area with respect to time presented a linear adjustment with a value of R^2^ of 0.9892 for *Canna hybrids* and 0.9795 for *Zantedeschia aethiopica*.

With the flowers produced by *Canna hybrids,* data had an average measure of 9.3 ± 0.8 cm, similar to that reported by Zamora-Castro et al. [39] and Cui et al. [40] for Canna flowers. With respect to *Z. aethiopica*, the average length of the flowers was similar to the 14 cm reported in the literature for plants cultivated in an experimental agricultural field during the same period of time. In addition, the number of flowers per plant falls within the range of flowers produced by this plant reported from 3 to 5 per year, applying compost to the crop [34]. This behavior of higher flower production in less time could be due to the presence of constant nutrients in the wastewater and the high elimination of phosphorus reported in this study (Table 2). With regard to the production of shoots (Figure 2f), this was higher for *Canna hybrids* than *Zantedeschia aethiopica.*, which may have been due to the fact that *Canna hybrids* is a promiscuous breeding plant.

**Figure 4:**
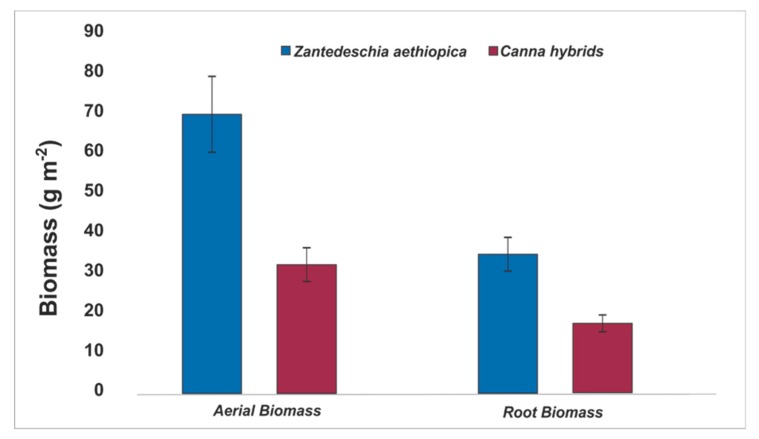
Aerial and subterranean biomass means of *Canna hybrids* and *Z. aethiopica* in partially saturated vertical CWs. Average ± standard deviation.

As for the biomass production for *Canna hybrids*, it was fast in VPS CWs. This species is suitable for use in CWs, for its rapid growth (Figure 2d) and its positive effect on the elimination of some specific pollutants (Table 3), although it is not a typical plant of natural wetlands. Figure 4 shows the biomass produced at the end of the study period, the dry matter obtained for both species showed a ratio of root biomass to aerial biomass of approximately 60:40, similar to that reported for soil cultivation of *Z. aethiopica* (60/40) [38]. The above indicates that VPS CWs can be a suitable growing system for this type of plant, allowing their use for commercial and/or aesthetic purposes in CW systems. Apparently, the partially saturated conditions of the CWs favored the development of these two species, taking into account that these conditions are more similar to those they have in their natural form of cultivation.

### 3.2. Wastewater Analysis

The optimum temperature for removing different contaminants in constructed wetlands is above 15 °C [41]. In this study, the temperature was measured at the input and output of the systems with a laboratory glass thermometer, and the average values are shown in Table 2. A significant decrease was found after passing through the mesocosms (*p* < 0.05) on average at 6 °C, which could be due to the hydraulic retention time used in this study, according to Akratos and Tsihrintzis [42].

According to Alemu et al. [43], the optimal pH to favor contaminant removal is in the range of 6.5–8.5, and the data reported in this study are in these ranges (Table 2). On the other hand, their behavior showed a significant increase after the treatment with the mesocosms (Table 2) (*p* < 0.05). This behavior can be explained by the capacity of the CWs to maintain approximately constant values (buffer) [44,45]. In relation to the OD, VPS CW mesocosms facilitate the diffusion of oxygen in the free-flow zones [13] (Figure 1). In the systems of this study, the presence of DO was high (Table 2), indicating aerobic conditions in the free drainage zone of the systems [13]. The systems with the presence of vegetation showed significant increases (*p* < 0.05) in relation to the systems without vegetation (Table 2); this could be due to the release of oxygen in the radical zone. However, this supply is very low and could be consumed quickly in the rhizosphere by aerobic microorganisms [46], which indicates that when taking the sample at the exit, oxygen could be increased.

### 3.3. Elimination of COD in CWs-VPS

COD is one of the main parameters used to measure the content of organic matter in wastewater and refers to the ability of the wastewater to deplete the content of dissolved oxygen [47]. Table 3 shows the removal of COD; no significant differences were found (*p* > 0.05) between systems planted with *Canna hybrids* and *Zantedeschia aethiopica* and without vegetation during the study period. These results are consistent with other studies that have found no significant differences between systems with and without vegetation [48]. On the other hand, in other studies with conventional CWs planted with *Canna hybrids* and *Zantedeschia ethiopica*, the removal efficiencies for COD have been found in the range of 40% to 70% with influent concentrations between 120 to 350 mg/L [49]. However, in this study, by using the same ornamental plants in novel VPS CWs (Table 3), the COD removal efficiencies were higher with also higher influent concentrations (517 to 584 mg/L). Apparently, the VPS CWs are more effective than other conventional CWs for COD removal; however, it is necessary to conduct a study with the same influent (and the same environmental conditions) to draw final conclusions.

### 3.4. Elimination of N-NH_4_ in CWs-VPS

The capacity of vertical flow CWs to carry out nitrification processes is well known, but in conditions of total saturation it is affected by the low presence of oxygen [13,27]. In this study, the presence of vegetation in the VPS CWs favored the elimination of N-NH_4_, in relation to the controls without vegetation (Table 3). Therefore, in the mesocosms with vegetation was eliminated more NH4^+^-N, with significant difference (*p* < 0.05) between the systems planted with *Canna hybrids* and *Zantedeschia aethiopica* but also between these and the controls without vegetation. The removals found in this study are superior to those reported by Zurita and White [50] in traditional vertical flow CWs planted with *Zantedeschia aethiopica* and superior to those reported by Zamora et al. [39] in vertical flow CWs using *Canna hybrids* and tezontle substrate. It appears that the elimination mechanisms in this study were probably, the adsorption on the substrate and the assimilation by the plants [20]. Therefore, regardless of the presence or absence of vegetation in the mesocosms, the vertical partially saturated CWs had greater ammonium eliminations than in other conventional vertical flow CWs, up to 15% in systems with vegetation and 12% in systems without vegetation. Table 4 shows the results of other studies with traditional vertical CWs, some of them with aeration, demonstrating that CWs-VPS show greater behavior in the elimination of N-NH_4_.

### 3.5. Elimination of N-NO_3_ in CWs-VPS

Denitrification is the main mechanism of elimination of NO_3_-N. It requires anoxic/anaerobic conditions (Figure 1), as well as a carbon source where facultative heterotrophic bacteria obtain energy by oxidizing organic matter [51], and it is limited by the presence of oxygen, pH, temperature, and supply of organic carbon [52]. The appropriate ranges for biological reactions in CWs are pH ranged between 6 to 8 [20,53], situations that prevailed in this study (Table 2). In terms of elimination results, no significant differences were found (*p* > 0.05) between the systems planted with *Canna hybrids* or *Zantedeschia aethiopica* (Table 3), but between the systems with and without vegetation (*p* < 0.05). Although the VPS CW systems have good oxygenation, due to the lower presence of oxygen in the saturated zone, these systems could make possible a greater elimination of nitrate. On the other hand, another mechanism for nitrate removal is the assimilation by plants (although plants prefer ammonium over nitrate) that in this study allowed the generation of high densities of tissues, corroborated with the biomass production (Figure 4) [10]. Additionally, in the mesocosms, greater removal was observed with rapid plant growth, so that this could be an indicator of the presence of high nutrient content in the plant tissues [20].

**Table 4:** Nitrogen removal in different studies with vertical flow CWs.

Scale	Type of Wetland	Plants	Pollutant Removal (%)	Reference
Microcosm	Subsurface Vertical Flow with Intermittent Aeration	*Oenanthe Javanica*	N-NH_4_: 15–28%. TN: 17–53%	Zhou et al. [54]
Microcosm	Subsurface Vertical Flow	*Phragmites australis*	N-NH_4_: 57–65%	Dan et al. [55]
Mesocosms	Subsurface Vertical Outdoor Flow with Modified Pallet Tanks	*A. halimus* *J. acutus* *S. perennis* *P. australis*	TN: 23–30%	Fountoulakis et al. [56]
Microcosm	Aerated Vertical Flow	*Acorus calamus L*	N-NH_4_: 43–81% TN: 29–52%	Zhang et al. [57]

### 3.6. Elimination of Norg in CWs-VPS

Facultative and/or anaerobic aerobic bacteria are responsible for the ammonification process, which is the main mechanism for the elimination of organic nitrogen in CWs [58]. In this study, removal efficiencies are shown in Table 3, finding significant differences (*p* < 0.05) between mesocosms with *Canna hybrids* and *Zantedeschia aethiopica* and without vegetation; the latter being lower than those planted with ornamental species. However, the results obtained are relatively higher than those reported in the literature in typical CWs [21,48]. Probably, these results were possible due to the greater presence of oxygen in the systems as well as the adequate pH values (Table 2) in the mesocosms; ammonification takes place in the range of 6.5 to 8.5 pH [59].

### 3.7. Elimination of TN in CWs-VPS

The results for total nitrogen removal in the mesocosms systems (Table 3) showed significant differences (*p* < 0.05) between systems with vegetation (similar results for both species) and without vegetation. The obtained results were superior to those found in 87 studies around the world with ornamental plants, reported by Sandoval et al. [49]. These results are explained by means of the nitrogen removal mechanisms in CWs, such as denitrification (in the saturated zone of the VPS CWs); assimilation by the plants, as demonstrated in their development (Figure 2d) and generation of remarkable biomass (Figure 4). Other processes were also possible, e.g., ammonification and nitrification (in the presence of oxygen in the free zone of the mesocosms and the plant radical zones). On the other hand, environmental conditions such as temperature and pH ranges favored the growth of bacteria (optimal ranges 6.6 to 8.0), which allowed an efficient elimination of NT in these systems. So that, different factors contributed to the high efficiencies of the mesocosms, such as the integration of free-flow and saturated conditions in the same system, the selection of an adequate substrate such as tezontle and the use of *Canna hybrids* and *Zantedeschia aethiopica* as emergent vegetation, for its easy adaptation and its rapid development in CWs.

### 3.8. TP Elimination in CWs-VPS

The results for total phosphorus removal are shown in Table 3. Significant differences were found (*p* < 0.05) between the systems with vegetation, being the elimination in the system with *Canna hybrids,* superior in 13% with respect to the system with *Zantedeschia aethiopica*. There were also significant differences (*p* < 0.05) between the systems with vegetation and those without vegetation; the systems with vegetation were more efficient in 35% to 55%. These results are high in relation to other studies as those reported by Shen et al. [60], who report removal ranges of 21% to 39% and Brix and Arias [61] who report 25% removals, both using stony substrates. On the other hand, the presence of vegetation played an important role in the removal, this could be due to the high production of flowers generated by plant species and biomass production, taking into account that phosphorus induces flowering in plants and the increase of biomass in them [20]. Ion exchange, precipitation in the systems [10] and the minerals contained in the teat could be another way of elimination [62].

## 4. Conclusions

VPS CWs proved to be more efficient than free-flow vertical CW systems for nitrogen removal; an increase in nitrogen removal in the 20–30% range is reported in the literature; with the additional advantages of reducing space to reach the same percentages of elimination of pollutants achieved in combined vertical and horizontal subsurface flow CWs.

The use of *Canna hybrids* and *Zantedeschia aethiopica* as emergent vegetation in VPS CWs is an alternative that increases the presence of dissolved oxygen in the systems; it also favors the elimination of specific compounds such as ammonium and phosphorus and additionally gives an aesthetic value to the systems facilitating their insertion in inter-urban and rural environments of areas where they are required for wastewater treatment.

Tropical weather conditions are favorable for the operation of these systems, given the higher temperatures, higher light intensity, and more standing vegetation that can be used in VPS CWs.

The results of plant development and biomass production indicate that the operating conditions of the VPS CWs favor the further development of plants and promote an environment of growth more similar to that which they may face in natural conditions of development. These multiple functions of the vegetation in the systems as phytoremediators and as ornamental plants that harmonize the landscape where they are implemented for the treatment of residual waters, and their use on a great scale can be with the aims of commercialize of this type of exotic plants that are developed in tropical and intertropical climates.

However, this study was performed only at the mesocosm scale, so that future studies should be implemented at a pilot scale (closer to large scales) to get more conclusive results with regard to the use of ornamental species with variations in design parameters, such as depth of saturated zones and free-flow zones in different climatic conditions and during longer periods of evaluation. In addition, other studies could focus on the evaluation of wastewater with a low carbon content that implies the addition of internal or external sources of carbon in the saturated zone of VPS CWs.

## Figures and Tables

**Table 1:** Chemical characteristics of wastewater used in this study.

Parameter	Value
COD (mg/L)	550.7 ± 33.6
N-NH_4_ (mg/L)	75.8 ± 21.7
N-NO_3_ (mg/L)	5.7 ± 2.4
N Org (mg/L)	8.3 ± 1.7
TN (mg/L)	99.8 ± 25.78
TP (mg/L)	9.7 ± 3.4
Dissolved Oxygen	1.2 ± 0.78
pH	8.0 ± 0.32

**Table 2:** Control parameters in the VPS CWs at a mesocosms scale.

Parameter	Influent	*Canna indica* + TZN	*Zantedeschia aethiopica* + TZN	Control TZN
Water Temperature (°C)	24.6 ± 2.3	18.3 ± 2.4	17.9 ± 1.4	19.1 ± 1.2
DO (mg/L)	1.2 ± 0.78	8.9 ± 0.3	7.2 ± 0.2	4.8 ± 0.4
pH	8.0 ± 0.32	7.4 ± 0.4	7.7 ± 0.2	7.05 ± 0.6

Values are given as the average ± standard deviation (*n* = 24). DO, dissolved oxygen; TZN, Tezontle.

**Table 3:** Wastewater quality parameters and pollutant removals in the mesocosms.

Parameters	Vegetation	Water Quality in the Mesocosms (Concentration mg/L)		Elimination Efficiency (%)
		Input	Output	
CDO (mg/L)	*Canna hybrids* + TZN	550.7 ± 33.6	16.4 ± 14.6	97.07 ± 2.72
	*Zantedeschia aethiopica* + TZN	550.7 ± 33.6	14.62 ± 11.4	97.47 ± 1.92
	Control TZN	550.7 ± 33.6	17.6 ± 11.6	96.92 ± 1.92
N-NH_4_ (mg/L)	*Canna hybrids* + TZN	75.8 ± 21.7	26.7 ± 12.6	72.52 ± 0.11
	*Zantedeschia aethiopica* + TZN	75.8 ± 21.7	33.5 ± 16.3	58.57 ± 9.64
	Control TZN	75.8 ± 21.7	52.4 ± 14.7	30.75 ± 0.43
N-NO_3_ (mg/L)	*Canna hybrids* + TZN	5.7 ± 2.4	1.1 ± 0.9	84.62 ± 9.32
	*Zantedeschia aethiopica* + TZN	5.7 ± 2.4	1.3 ± 1.1	82.44 ± 9.5
	Control TZN	5.7 ± 2.4	0.3 ± 0.2	94.40 ± 1.57
N Org (mg/L)	*Canna hybrids* + TZN	8.3 ± 1.7	2.1 ± 1.5	77.46 ± 13.46
	*Zantedeschia aethiopica* + TZN	8.3 ± 1.7	2.7 ± 2.1	71.38 ± 19.45
	Control TZN	8.3 ± 1.7	3.6 ± 1.4	58.34 ± 8.34
TN (mg/L)	*Canna hybrids* + TZN	99.8 ± 25.78	29.9 ± 14.87	72.02 ± 7.67
	*Zantedeschia aethiopica* + TZN	99.8 ± 25.78	37.5 ± 19.49	65.15 ± 10.52
	Control TZN	99.8 ± 25.78	56.3 ± 16.26	44.07 ± 1.84
TP (mg/L)	*Canna hybrids* + TZN	9.7 ± 3.4	0.3 ± 0.2	95.30 ± 0.89
	*Zantedeschia aethiopica* + TZN	9.7 ± 3.4	2.1 ± 1.6	81.89 ± 10.17
	Control TZN	9.7 ± 3.4	5.6 ± 1.8	41.6 ± 1.91

Values are given as the average ± standard deviation (*n* = 24).

## References

[1] Friedler E., Butler D., Alfiya Y. (2013). Source Separation and Decentralization Wastewater Management.

[2] Holmes D.E., Dang Y., Smith J.A. (2019). Nitrogen cycling during wastewater treatment. Adv. Appl. Microbiol..

[3] Chen D., Gu X., Zhu W., He S., Huang J., Zhou W. (2019). Electrons transfer determined greenhouse gas emissions in enhanced nitrogen-removal constructed wetlands with different carbon sources and carbon-to-nitrogen ratios. Bioresour. Technol..

[4] Chang M., Wang Y., Pan Y., Zhang K., Lyu L., Wang M., Zhu T. (2019). Nitrogen removal from wastewater via simultaneous nitrification and denitrification using a biological folded non-aerated filter. Bioresour. Technol..

[5] Aldaya M.M., Rodriguez C.I., Fernandez-Poulussen A., Merchan D., Beriain M.J., Llamas R. (2019). Grey water footprint as an indicator for diffuse nitrogen pollution: The case of Navarra, Spain. Sci. Total Environ..

[6] Ghimire U., Nandimandalam H., Martinez-Guerra E., Gude V.G. (2019). Wetlands for Wastewater Treatment. Water Environ. Res..

[7] Shi W., Li H., Li A. (2018). Mechanism and influencing factors of nitrogen removal in subsurface flow constructed wetland. Appl. Chem. Eng..

[8] Kumar S., Dutta V. (2019). Constructed wetland microcosms as sustainable technology for domestic wastewater treatment: An overview. Environ. Sci. Pollut. Res..

[9] Tchobanoglous G., Burton F.L., Stensel H.D. (2003). Metcalf & Eddy wastewater engineering: Treatment and reuse. Int. Edition. McGrawHill.

[10] Bojorges T., Xitlalli Á., Hernández Razo N.A., Urquieta F., Aseret A., Zurita Martínez F. (2017). Evaluación de tres sistemas de humedales híbridos a escala piloto para la remoción de nitrógeno. Rev. Int. Cont. Amb..

[11] Avellán T., Gremillion P. (2019). Constructed wetlands for resource recovery in developing countries. Renew. Sustain. Energy Rev..

[12] Aalam T., Khalil N. (2019). Performance of horizontal sub-surface flow constructed wetlands with different flow patterns using dual media for low-strength municipal wastewater: A case of pilot scale experiment in a tropical climate region. J. Environ. Sci. Health Part A.

[13] Martínez N.B., Tejeda A., Del Toro A., Sánchez M.P., Zurita F. (2018). Nitrogen removal in pilot-scale partially saturated vertical wetlands with and without an internal source of carbon. Sci. Total Environ..

[14] Ali Z., Mohammad A., Riaz Y., Quraishi U.M., Malik R.N. (2018). Treatment efficiency of a hybrid constructed wetland system for municipal wastewater and its suitability for crop irrigation. Int. J. Phytoremed..

[15] Herrera-Melián J., Borreguero-Fabelo A., Araña J., Peñate-Castellano N., Ortega-Méndez J. (2018). Effect of Substrate, Feeding Mode and Number of Stages on the Performance of Hybrid Constructed Wetland Systems. Water.

[16] Li H., Liu F., Luo P., Chen X., Chen J., Huang Z., Peng J., Xiao R., Wu J. (2019). Stimulation of optimized influent C: N ratios on nitrogen removal in surface flow constructed wetlands: Performance and microbial mechanisms. Sci. Total Environ..

[17] Groh T.A., Gentry L.E., David M.B. (2015). Nitrogen removal and greenhouse gas emissions from constructed wetlands receiving tile drainage water. J. Environ. Qual..

[18] Mitsch W.J., Gosselink J. (2015). Wetlands.

[19] Saeed T., Sun G. (2012). A review on nitrogen and organics removal mechanisms in subsurface flow constructed wetlands: Dependency on environmental parameters, operating conditions and supporting media. J. Environ. Manag..

[20] Vymazal J. (2007). Removal of nutrients in various types of constructed wetlands. Sci. Total Environ..

[21] Ilyas H., Masih I. (2017). The performance of the intensified constructed wetlands for organic matter and nitrogen removal: A review. J. Environ. Manag..

[22] Lin-Lan Z., Ting Y., Jian Z., Xiangzheng L. (2019). The configuration, purification effect and mechanism of intensified constructed wetland for wastewater treatment from the aspect of nitrogen removal: A review. Bioresour. Technol..

[23] Vymazal J. (2013). The use of hybrid constructed wetlands for wastewater treatment with special attention to nitrogen removal: A review of a recent development. Water Res..

[24] Srivastava P., Yadav A.K., Garaniya V., Lewis T., Abbassi R., Khan S. (2019). Electrode dependent anaerobic ammonium oxidation in microbial fuel cell integrated hybrid constructed wetlands: A new process. Sci. Total Environ..

[25] Silveira D.D., Belli Filho P., Philippi L.S., Kim B., Molle P. (2015). Influence of partial saturation on total nitrogen removal in a single-stage French constructed wetland treating raw domestic wastewater. Ecol. Eng..

[26] Kraiem K., Kallali H., Wahab M.A., Fra-vazquez A., Mosquera-Corral A., Jedidi N. (2019). Comparative study on pilots between ANAMMOX favored conditions in a partially saturated vertical flow constructed wetland and a hybrid system for rural wastewater treatment. Sci. Total Environ..

[27] Han Z., Miao Y., Dong J., Shen Z., Zhou Y., Liu S., Yang C. (2019). Enhanced nitrogen removal and microbial analysis in partially saturated constructed wetland for treating anaerobically digested swine wastewater. Front. Environ. Sci. Eng..

[28] National Institute of Statistical Geography and Data Processing (2014). Yearbook Statistical and Geographical of Veracruz de Ignacio de la Llave. http://www.inegi.gob.mx.

[29] Zurita F., de Anda J., Belmont M.A. (2006). Performance of laboratory-scale wetlands planted with tropical ornamental plants to treat domestic wastewater. Water Qual. Res. J..

[30] Trejo-Téllez L.I., Ramírez-Martínez M., Gómez-Merino F.C., García-Albarado J.C., Baca-Castillo G.A., Tejeda-Sartorius O. (2013). Physical and chemical evaluation of volcanic rocks and its use for tulip production. Rev. Mex. Cienc. Agrícolas.

[31] Marín-Muñiz J.L., García-González M.C., Ruelas-Monjardín L.C., Moreno-Casasola P. (2018). Influence of different porous media and ornamental vegetation on wastewater pollutant removal in vertical subsurface flow wetland microcosms. Environ. Eng. Sci..

[32] American Public Health Association (APHA) (2005). Standard Methods for the Examination of Water and Wastewater.

[33] Montgomery D.C. (2017). Design and Analysis of Experiments.

[34] Cruz-Castillo J.G., Torres-Lima P.A. (2017). ‘Deja Vu’: A new calla lily (*Zantedeschia aethiopica*) cultivar. Rev. Chapingo Ser. Hortic..

[35] Haritash A.K., Sharma A., Bahel K. (2015). The Potential of Canna lily for Wastewater Treatment Under Indian Conditions. Int. J. Phytoremed..

[36] Tran H.D., Vi H.M.T., Dang H.T.T., Narbaitz R.M. (2019). Pollutant removal by Canna Generalis in tropical constructed wetlands for domestic wastewater treatment. Glob. J. Environ. Sci. Manag..

[37] Kato M., Inthavongsa K., Imai K. (1989). An estimation of leaf area in edible canna (*Canna edulis Ker.*). Jpn. J. Crop. Sci..

[38] Casierra-Posada F., Nieto P.J., Ulrichs C. (2012). Crecimiento, producción y calidad de flores en calas (*Zantedeschia aethiopica* (L.) K. Spreng) expuestas a diferente calidad de luz. Rev. UDCAv Div. Cient..

[39] Zamora-Castro S.A., Marín-Muñiz J.L., Sandoval L., Vidal-Álvarez M., Carrión-Delgado J.M. (2019). Effect of Ornamental Plants, Seasonality, and Filter Media Material in Fill-and-Drain Constructed Wetlands Treating Rural Community Wastewater. Sustainability.

[40] Cui L., Ouyang Y., Lou Q., Yang F., Chen Y., Zhu W., Luo S. (2012). Removal of nutrients from wastewater with Canna indica L. under different vertical-flow constructed wetland conditions. Ecol. Eng..

[41] Kuschk P., Wiessner A., Kappelmeyer U., Weissbrodt E., Kästner M., Stottmeister U. (2003). Annual cycle of nitrogen removal by a pilot-scale subsurface horizontal flow in a constructed wetland under moderate climate. Water Res..

[42] Akratos C.S., Tsihrintzis V.A. (2007). Effect of temperature, HRT, vegetation and porous media on removal efficiency of pilot-scale horizontal subsurface flow constructed wetlands. Ecol. Eng..

[43] Alemu K., Assefa B., Kifle D., Kloos H. (2018). Nitrogen and Phosphorous Removal from Municipal Wastewater Using High Rate Algae Ponds. INAE Lett..

[44] Kadlec R.H. (2009). Comparison of free water and horizontal subsurface treatment wetlands. Ecol. Eng..

[45] Winkler M.K., Straka L. (2019). New directions in biological nitrogen removal and recovery from wastewater. Curr. Opin. Biotechnol..

[46] Wiebner A., Kappelmeyer K., Kuschk P., Kästner M. (2005). Influence of the redox condition dynamics on the removal efficiency of a laboratory-scale constructed wetland. Water Res..

[47] Zhang X., Zha L., Jiang P., Wang X., Lu K., He S., Huang J., Zhou W. (2019). Comparative study on nitrogen removal and functional genes response between surface flow constructed wetland and floating treatment wetland planted with Iris pseudacorus. Environ. Sci. Pollut. Res..

[48] Li X., Zhang M., Liu F., Chen L., Li Y., Xiao R., Wu J. (2018). Seasonality distribution of the abundance and activity of nitrification and denitrification microorganisms in sediments of surface flow constructed wetlands planted with Myriophyllum elatinoides during swine wastewater treatment. Bioresour. Technol..

[49] Sandoval L., Zamora-Castro S.A., Vidal-Álvarez M., Marín-Muñiz J.L. (2019). Role of Wetland Plants and Use of Ornamental Flowering Plants in Constructed Wetlands for Wastewater Treatment: A Review. Appl. Sci..

[50] Zurita F., White J. (2014). Comparative study of three two-stage hybrid ecological wastewater treatment systems for producing high nutrient, reclaimed water for irrigation reuse in developing countries. Water.

[51] Saggar S., Jha N., Deslippe J., Bolan N.S., Luo J., Giltrap D.L., Kim D.-G., Zaman M., Tillman R.W. (2013). Denitrification and N_2_O: N_2_ production in temperate grasslands: Processes, measurements, modelling and mitigating negative impacts. Sci. Total Environ..

[52] Vera L., Vidal G., Salvato M., Borin M. (2011). Consideraciones para la eliminación del nitrógeno en humedales artificiales. Tecnol. Agua.

[53] Dušek J., Picek T., Čížková H. (2008). Redox potential dynamics in a horizontal subsurface flow constructed wetland for wastewater treatment: Diel, seasonal and spatial fluctuations. Ecol. Eng..

[54] Zhou X., Wang X., Zhang H., Wu H. (2017). Enhanced nitrogen removal of low C/N domestic wastewater using a biochar-amended aerated vertical flow constructed wetland. Bioresour. Technol..

[55] Dan A., Fujii D., Soda S., Machimura T., Ike M. (2017). Removal of phenol, bisphenol A, and 4-tert-butylphenol from synthetic landfill leachate by vertical flow constructed wetlands. Sci. Total Environ..

[56] Fountoulakis M.S., Sabathianakis G., Kritsotakis I., Kabourakis E.M., Manios T. (2017). Halophytes as vertical-flow constructed wetland vegetation for domestic wastewater treatment. Sci. Total Environ..

[57] Zhang X., Hu Z., Ngo H.H., Zhang J., Guo W., Liang S., Xie H. (2018). Simultaneous improvement of waste gas purification and nitrogen removal using a novel aerated vertical flow constructed wetland. Water Res..

[58] Arteaga-Cortez V.M., Quevedo-Nolasco A., del Valle-Paniagua D.H., Castro-Popoca M., Bravo-Vinaja Á., Ramírez-Zierold J.A. (2019). A current review of the mechanisms that make the artificial wetlands for the removal of nitrogen and phosphorus. Tecnol. Cienc. Agua.

[59] Zurita F., De Anda J., Belmont M.A. (2009). Treatment of domestic wastewater and production of commercial flowers in vertical and horizontal subsurface-flow constructed wetlands. Ecol. Eng..

[60] Shen Y., Zhuang L., Zhang J., Fan J., Yang T., Sun S. (2019). A study of ferric-carbon micro-electrolysis process to enhance nitrogen and phosphorus removal efficiency in subsurface flow constructed wetlands. Chem. Eng. J..

[61] Brix H., Arias C.A. (2005). The use of vertical flow constructed wetlands for on-site treatment of domestic wastewater: New Danish guidelines. Ecol. Eng..

[62] Bolton L., Joseph S., Greenway M., Donne S., Munroe P., Marjo C.E. (2019). Phosphorus adsorption onto an enriched biochar substrate in constructed wetlands treating wastewater. Ecol. Eng..

